# Surgical treatment of lumbar spinal stenosis with microdecompression and interspinous distraction device insertion. A case series

**DOI:** 10.1186/1749-799X-7-35

**Published:** 2012-10-29

**Authors:** Avraam Ploumis, Pavlos Christodoulou, Dimitrios Kapoutsis, Ioannis Gelalis, Vasilios Vraggalas, Alexander Beris

**Affiliations:** 1 Departments of Orthopaedics and Rehabilitation, University of Ioannina, Ioannina, Greece; 2 Orthopaedic Department, 424 General Army Hospital, Thessaloniki, Greece

**Keywords:** IPDD, X-STOP, Microdecompression, Spinal stenosis

## Abstract

**Background:**

Interspinous distraction devices (IPDD) are indicated as stand-alone devices for the treatment of spinal stenosis. The purpose of this study is to evaluate the results of patients undergoing surgery for spinal stenosis with a combination of unilateral microdecompression and interspinous distraction device insertion.

**Methods:**

This is a prospective clinical and radiological study of minimum 2 years follow-up. Twenty-two patients (average age 64.5 years) with low-back pain and unilateral sciatica underwent decompressive surgery for lumbar spinal stenosis. Visual Analogue Scale, Oswestry Disability Index and walking capacity plus radiologic measurements of posterior disc height of the involved level and lumbar lordosis Cobb angle were documented both preoperatively and postoperatively. One-sided posterior subarticular and foraminal decompression was conducted followed by dynamic stabilization of the diseased level with an IPDD (X-STOP).

**Results:**

The average follow-up time was 27.4 months. Visual Analogue Scale and Oswestry Disability Index improved statistically significantly (p < 0.001) in the last follow-up exam. Also, the walking distance increased in all patients but two. Posterior intervertebral disc height of the diseased level widened average 1.8 mm in the postoperative radiograph compared to the preoperative. No major complication, including implant failure or spinous process breakage, has been observed.

**Conclusions:**

The described surgical technique using unilateral microdecompression and IPDD insertion is a clinically effective and radiologically viable treatment method for symptoms of spinal stenosis resistant to non-operative treatment.

## Background

Lumbar spinal stenosis, refractory to nonoperative treatment, has been traditionally treated with surgical decompression achieving good and excellent results in almost 80% of the cases [1]. In case of coexisting instability or deformity or discogenic pain, fusion is added. However, the immobilization of a spinal unit causes significant functional changes to the patients in terms of spinal mobility and symptomatology originating from adjacent segments [2].

“Dynamic stabilization” spinal system is defined as the system that alters favorably the biomechanics (movement and load transmission) of a spinal motion segment, without immobilization of the segment [3,4]. X-STOP is an interspinous distraction device (IPDD) leading to indirect decompression by distraction [5] and disc unloading [6]. It is indicated for patients older than 50 years old with symptomatology of neurogenic claudication and radiographic spinal stenosis up to two levels. It has been suggested to be superior to laminectomy with or without fusion for the above indications offering minimal surgical trauma, avoidance of bone removal, immediate symptom relief, fast recovery and rehabilitation and low complication rate [7-9].

This is the first usage of X-STOP (by St. Francis Medical Technologies, Inc. Alameda, CA and, lately, by Medtronic Inc., Minneapolis, MN) device, an FDA approved IPDD for patients with spinal stenosis [10], in conjunction with minimally invasive lateral decompression [11]. The purpose of this study is to present the two-year follow-up results of surgical treatment for patients with symptomatology of low-back pain and unilateral sciatica due to spinal stenosis who did not respond to nonoperative treatment. Our hypothesis is that the combination of mini-open lateral decompression and X-STOP insertion causes clinical improvement of symptomatology without lordosis deterioration. Since no control group was used, this mode of treatment is compared to outcomes reported in studies using X-STOP alone for lumbar spinal stenosis.

## Methods

This a prospective study of 22 patients aged between 57–71 years (average 64.5 years) with unilateral sciatica and low-back pain, in the form of neurogenic intermittent claudication, who were treated by unilateral microdecompression and X-STOP insertion in our hospital between 2006–2007. All patients consented to participate in the study and ethics committee approval (no 11/2005 424 General Army Hospital, Thessaloniki, Greece) for the study was obtained. The average T-score of bone density measurement (DEXA) studies of the aforementioned patients was −1.9. Patients’ activities involved mainly household duties, gardening and daily walking but the disease significantly affected their quality of life. All patients had plain x-rays in standing position, dynamic radiographs in flexion extension and MRI of the lumbar spine. No patient had osteoporosis (T-score>−2.5), more than grade I spondylolisthesis, dynamic instability, motor deficit or severe lumbar spinal stenosis (with or without kissing spinous processes)as these were exclusion criteria in our study and containdications for X-STOP insertion. All patients had both central and lateral spinal stenosis (subarticular, foraminal or both) with disc degeneration at the involved levels.

The degree of spinal stenosis, according to radiologist reading of the MRI, was mild to moderate. For central stenosis, dural sac cross sectional area had to be between 100 and 130mm2 in the central canal area of transverse MRI. Less than 100mm2 was considered severe stenosis and these cases were excluded [12]. Mild to moderate subarticular stenosis was defined as contact or deviation of nerve root in the transverse cut MRI but cases with root deformation were excluded [13]. Finally, cases with foraminal stenosis were included if the epidural fat was shown to be disappearing only partially in the sagittal plane MRI [14].

In all cases pathology was found at L4-L5 level except for four patients with stenosis both at L3-L4 and L4-L5 levels. Initially nonoperative treatment for at least 3 months was applied for all patients including non-steroidal anti-inflammatories, physical therapy, bracing and transforaminal epidural steroid injections/selective nerve root injections. When nonoperative treatment did not alleviate their symptoms, surgical treatment was performed. Microdecompression of the subarticular and foraminal stenosis was based on the responses from selective nerve root injections with anesthetic and steroids.

The Oswestry Disability Index (ODI) and the Visual Analogue Scale (VAS) (for low-back pain and leg pain separately) scores were documented preoperatively, at the third week, six months and first year postoperatively and at the last follow-up. Self-rated walking distance preoperatively and after surgery at last follow up as well as satisfaction regarding overall result of back operation, according to questions of Zurich Questionnaire, were evaluated [15].

Also, segmental lordosis of the involved levels and lumbar lordosis (upper plate of L1 to upper plate of S1) were measured by the Cobb angle in standing x-rays both preoperatively and at the last follow-up. Finally, radiographic posterior disc height of the involved levels was measured preoperatively and at the last follow-up. Radiographic manual measurements were done twice by the first two authors (AP, PC) and the average was recorded.

Statistical analysis both for the functional and radiographic results was performed with the use SSPS 10.0. The comparison was calculated with Student’s paired t-test. Statistical significance was considered when p < 0.05.

### Surgical technique

Under general anesthesia and prone position on a radiolucent table with padded support at the level of the iliac crests and sternum, slight flexion of hips and knees was preserved so that the subjects lie in a less lordotic position. After an appropriately small skin incision (approximately 4 centimeters), under fluoroscopic control, and subcutaneous tissue incision, the dissection went through the dorsolumbar fascia approximately 5–10 mm lateral to the midline towards the symptomatic side, preserving the supraspinous ligamentous attachment to the fascia. The multifidus was detached from the symptomatic side of the spinous processes and laminar attachments. Retractors were placed to keep the wound open and were being loosened regularly to avoid damage to the retracted muscles. The ligamentum flavum was detached with a freer elevator and then completely resected. The superior and inferior laminae were only partially resected. Decompression of both exiting and transversing nerve roots at that level were performed under microscope magnification. Partial facetectomies (mainly undercutting the superior facet) and foraminal decompressions were carried out with the aid of angled Kerrisson rongeurs of varying widths and lengths and/or a power drill. The adequacy of decompression was checked with foraminal probes. No removal of the central portion of laminae and no discectomy were performed.

Next, the fascia was split longitudinally also to the other, non-decompression, side of midline and multifidus is detached only next to the interspinous area. It was of paramount importance for the stability of the segment to keep the supraspinous and part of the interspinous ligaments intact [16]. A small and, then, a larger blunt curved dilator were inserted across the interspinous space abutting the posterior border of the facet joints at the most anterior margin of the interspinous space. After the larger dilator was removed, a sizing distraction instrument is inserted. The correct implant size was indicated on the sizing instrument by tightening the supraspinous ligament in distraction. An appropriately sized X-STOP device was inserted between the spinous processes until it was flush with the right side of the spinous processes and as much anteriorly as possible in the interlaminar space. The screw hole for the universal wing on the left side was visualized and the universal wing screw was engaged. The two wings were approximated toward the midline and the left-sided universal wing screw was secured with a torque-limiting hexagonal screwdriver. Anteroposterior and lateral fluoroscopy views were taken to verify the proper position.

After completion of both thorough decompression and X-STOP insertion, the dorsolumbar fascia was sutured over a suction drain.

## Results

The follow-up period ranged from 24 to 36 months and averaged 27.4 months. All patients were able to return to their daily activities within six weeks postoperatively. Both ODI and VAS low-back and leg pain scores improved significantly postoperatively (p < 0.001) and this improvement deteriorated slightly (p > 0.05) in the last follow-up (Table 1). Using a 25-point improvement from baseline Oswestry Disability Index score as a success criterion, 20 out of 22 patients (91%) had successful outcomes.

**Table 1:** Clinical and radiographic measurements (mean (SD))

	**Preoperatively**	**Last follow-up**	**p-value**
ODI	50.7 (4.4)	19.9 (3.7)	< 0.001
VAS (back pain)	4.5 (1.0)	2.5 (0.7)	< 0.001
VAS (leg pain)	7.5 (0.6)	2.6 (0.8)	< 0.001
Posterior intervertebral height (mm)	5.9 (1.6)	7.7 (2.2)	< 0.001
Lumbar lordosis (°)	52.8 (4.5)	53.4 (4.4)	non-significant
Segmental lordosis (°)	5.7 (3.5)	3.3 (2.5)	< 0.001

**Figure 1:**
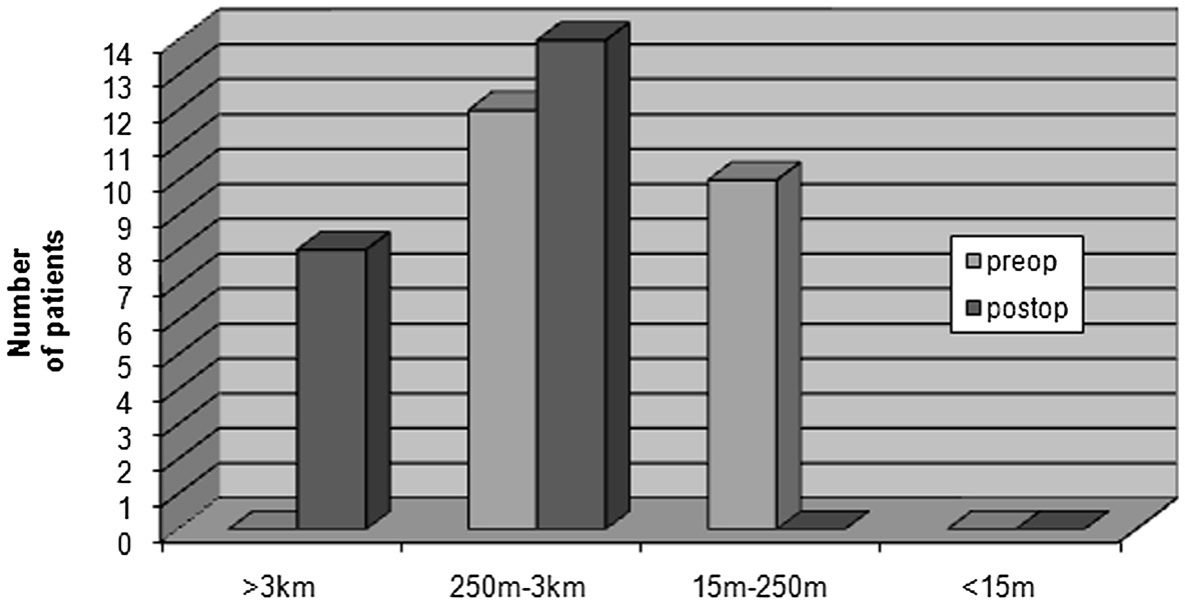
Chart showing preoperative and postoperative walking capacity of patients according to Zurich Questionnaire.

When the self-assessment evaluation of Zurich Questionnaire was used for the satisfaction from surgery, only two patients responded somewhat satisfied while the rest twenty patients (90.9%) declared very satisfied. In terms of walking distance, none deteriorated, six patients improved one grade and four improved two grades (Figure 1).

The interspinous implants used were mainly 10mm and 12mm height size and 8mm implant was inserted only in two levels.

**Figure 2:**
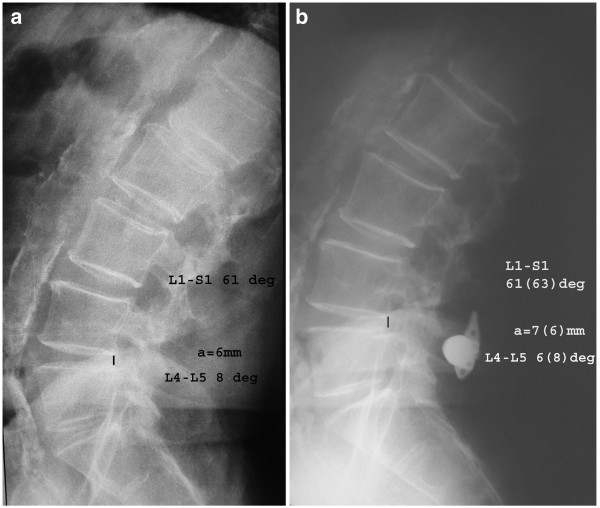
Preoperative (A) and postoperative (B) lateral lumbar radiograph of a patient treated with x-stop insertion and left direct lateral decompression showing increase of posterior intervertebral disc height and preservation of lumbar lordosis.

Radiographic analysis of standing x-rays of the lumbar spine showed a mean increase of 1.8 mm of the posterior intervertebral disc height of the operated levels and the postoperative height was significantly larger than preoperatively (p < 0.001) (Figure 2). Specifically, there was an increase of posterior disc height after implant insertion in 23 out of 26 disc spaces. Also, the mean segmental lordosis was significantly decreased (p < 0.001) postoperatively compared to preoperative respective value. However, the mean lumbar lordosis did not show statistical significant change (p > 0.05) in the postoperative period (Table 1).

**Figure 3:**
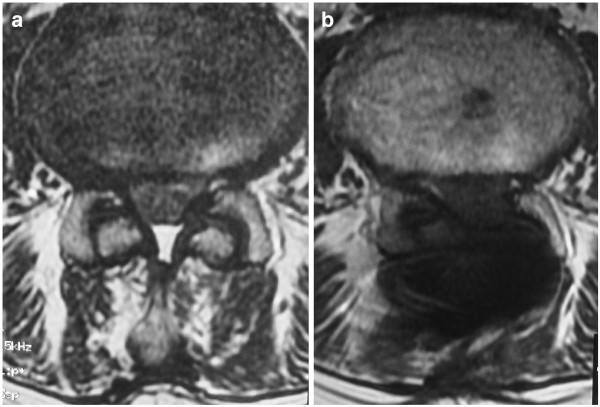
**Preoperative (A) and postoperative (B) transverse T2 MRI images at the stenotic level of the same patient shown in Figure**2**.** Unilateral microdecompression and X-STOP insertion was performed. The postoperative increase of the space available for the sac is shown.

In a pilot study of our first patients, the postoperative MRI showed the expansion of the space available for the sac following the unilateral decompression (Figure 3).

There were no major complications in our series. No obvious by plain x-rays spinous process fracture was detected; however there was one asymptomatic case of gradual displacement of the superior to the implant spinous process which suggested a possible fracture. Two patients with radiculitis originating from the level above the decompressed level were treated successfully with anti-inflammatory medication five months postoperatively. In another two cases, the X-STOP device was not inserted anteriorly enough in the interspinous space but this did not cause symptoms.

## Discussion

The rationale for this study was to evaluate if the combination of direct unilateral decompression and the indirect decompression with an IPDD device (X-STOP) was effective for the patients with neurogenic claudication due to spinal stenosis and, also, to compare our results with historical controls of other studies using only X-STOP device. This study intends to show the validity of the presented method of direct and indirect decompression of neural elements and not to show the superiority to other established treatment methods (stand-alone decompression, stand-alone X-STOP insertion).

The first IPDD implanted in patients with neurogenic intermittent claudicaton (NIC) due to spinal stenosis was the X-STOP (first FDA approved) [3]. X-STOP insertion with blunt instruments only does not violate the supraspinous/interspinous complex and can be implanted under local anesthesia [17].

X-STOP reduces facet load and prevents narrowing of the canal and foramen [18-20]. As Cabraja et al. described [5], insertion of an interspinous device can decrease the facet joint back pain. Similarly, patients in our study showed significant less axial low-back pain postoperatively than preoperativiely. However, the main benefit was the decrease of leg pain in the form of neurogenic caludication.

In cases with lateral spinal stenosis including subarticular and foraminal nerve root impingement, indirect decompression by X-STOP insertion only may not be as beneficial when there is long-term inflammation in the area. It is advantageous that a direct decompression is performed in lateral subarticular and foraminal area in order to free nerve roots from adhesions due to the chronic inflammation tissue. This combination of direct (mini-open) and indirect (by X-STOP insertion) decompression was performed in our patients. In a biomechanical cadaveric study of X-STOP device by Fuchs et al. [21], unilateral facetectomies did not lead to added instability of the spinal unit with interspinous devices and in contrast to the instability caused by bilateral facetectomies.

The addition of X-STOP device can distract the intervertebral space and preserve the nerve-occupying space without sacrificing the mobility of the motion segment [7-9].

In Table 2, there is a list of bibliographic studies with patients with spinal stenosis and neurogenic claudication treated surgically with X-STOP only without direct decompression. The patients of our study achieved similar improvement in functional scores and higher satisfaction (except Kuchta study [22]) in the second postoperative year compared to patients treated with X-STOP only.

**Table 2:** Bibliographic analysis of clinical outcome studies with x-stop insertion for spinal stenosis

**Study**	**# of pts**	**Type of surgery**	**f-u time (months)**	**Mean outcomes improvement at last follow-up**	**Patient satisfaction domain of ZCQ (% of pts)**
Kondrashov et al (JSDT 2006) [14]	18	x-stop only	51	29 (ODI)	not reported
Shidiqui et al (Spine 2007) [22]	24	x-stop only	12	11 (ODI)	71
Zucherman et al (Spine 2005) [8]	93	x-stop only	24	45.4% (ZCQ symptom severity domain)	73.1
Brussee et al (Eur Spine J 2008) [23]	65	x-stop only	12	48.4% (ZCQ walking ability)	74.2
Kuchta et al (Eur Spine J 2009) [20]	175	x-stop only	24	12 (ODI)	not reported
Ploumis et al	22	x-stop plus decompression	27.4	30 (ODI)	90.9

In a randomized controlled, prospective, multicenter trial outcome study, patients suffering from neurogenic intermittent claudication treated with X-STOP were compared to outcomes of similargroup patients treated conservatively [9,10]. These class I clinical data included patients with mild to moderate spinal stenosis. X-STOP was found significantly more effective compared to conservative treatment after two years follow up. The X-STOP patients group had significant improvement of clinical symptoms and function compared with epidural steroid injections treated patient group. Successful outcome in our unicenter study group, even though in smaller numbers, reached to 91% of the patients.

Lee et al [23]. experience similar satisfaction rates with Zucherman et al [10],. but the individuals in his study found only 40% improvement in severity of symptoms.

29% of the patients in Siddiqui et al. study [24] lost beneficial effect of X-STOP implantation after the third month and had to have epidural steroid injection in a year’s time after the operation. It was presumed that because of the position of the device, bony indentations occur at the contact area of the spinous process and with time this led to reduction of the initial distraction. Kim et al. reported postoperative fractures of the spinous processes diagnosed by CT in 22% of patients with IPDD insertion but only half of them were symptomatic [25]. All of our patients had well seated implants in the anterior interspinous area, except two who did not need revision.

In our study elective microdecompression surgery was combined with X-STOP insertion. The patients included were suffering from mild to moderate severity central and unilateral lumbar spinal stenosis. Conservative treatment had been followed for at least 6 months with no relieving response. The procedure included partial unilateral laminotomy, subarticular and foraminal (undercutting) decompression with an effort to avoid complete facetectomies. After microdecompression, X-STOP was applied at the diseased levels. All the patients were evaluated before surgery and postoperatively at 3 weeks, 6 months, 1 year and 2 years follow-up. Our outcome tools were VAS, ODI and certain questions on walking capacity and patient satisfaction from Zurich Questionnaire, all of which improved significantly. Posterior disc height of the involved level and Cobb angle were documented pre- and postoperatively. Lumbar lordotic Cobb angle did not change statistically significantly even though segmental lordosis decreased. This means that lordosis increased in the adjacent motion segments to improve sagittal balance. In contrary to other studies, posterior intervertebral disc height at the involved levels increased in average 1.8 mm postoperatively compared to preoperatively in accordance to results from Sobottke et al [26]. No major complication, significant blood loss, or slippage of device was detected.

The anatomic advantage of direct decompression of neural tissues is obviously superior to indirect decompression. The financial cost of adding an IPDD in a microdecompression case may be exchanged by the lasting clinical improvement and the preservation of disc height and lordosis.

Limitation to our study is the absence of a control patient group. A prospective randomized study comparing IPDD insertion only to IPDD insertion plus direct decompression is under way. This study provides level IV evidence and should be considered this way.

## Conclusions

Direct lateral microdecompression in addition to indirect decompression by an IPDD (X-STOP) insertion is a clinically effective and radiologically viable treatment option for patients with mild to moderate lumbar spinal stenosis.

## Competing interests

There is no personal or institutional financial support related to the study.

## Authors’ contributions

AP conceived of the study, performed the statistical analysis and drafted the manuscript. PC, DK, IG, VV, AB participated in its design and coordination and helped to draft the manuscript. All authors read and approved the final manuscript.

## References

[B1] Sengupta DK Herkowitz HN Lumbar spinal stenosis. Treatment strategies and indications for surgery Orthop Clin North Am 2003 34 281 295 10.1016/S0030-5898(02)00069-X 12914268

[B2] Thomsen K Christensen FB Eiskjaer SP Hansen ES Fruensgaard S Bunger CE Volvo Award winner in clinical studies. The effect of pedicle screw instrumentation on functional outcome and fusion rates in posterolateral lumbar spinal fusion: a prospective, randomized clinical study Spine 1997 22 1997 2813 2822 9431617 10.1097/00007632-199712150-00004

[B3] Christie SD Song JK Fessler RG Dynamic interspinous process technology Spine 2005 30 S73 S78 10.1097/01.brs.0000174532.58468.6c 16103838

[B4] Sengupta DK Dynamic stabilization devices in the treatment of low back pain Orthop Clin North Am 2004 35 43 56 10.1016/S0030-5898(03)00087-7 15062717

[B5] Cabraja M Abbushi A Woiciechowsky C Kroppenstedt S The short- and mid-term effect of dynamic interspinous distraction in the treatment of recurrent lumbar facet joint pain Eur Spine J 2009 18 1686 1694 10.1007/s00586-009-1149-8 19727852 PMC2899396

[B6] Lindsey DP Swanson KE Fuchs P Hsu KY Zucherman JF Yerby SA The effects of an interspinous implant on the kinematics of the instrumented and adjacent levels in the lumbar spine Spine 2003 28 2192 2197 10.1097/01.BRS.0000084877.88192.8E 14520030

[B7] Katz JN Lipson SJ Chang LC Levine SA Fossel AH Liang MH Seven- to 10-year outcome of decompressive surgery for degenerative lumbar spinal stenosis. Spine 1996 21 92 98 10.1097/00007632-199601010-00022 9122770

[B8] Katz JN Stucki G Lipson SJ Fossel AH Grobler LJ Weinstein JN Predictors of surgical outcome in degenerative lumbar spinal stenosis Spine 1999 24 2229 2233 10.1097/00007632-199911010-00010 10562989

[B9] Zucherman JF Hsu KY Hartjen CA Mehalic TF Implicito DA Martin MJ Johnson DR 2nd Skidmore GA Vessa PP Dwyer JW Puccio S Cauthen JC Ozuna RM A prospective randomized multi-center study for the treatment of lumbar spinal stenosis with the X STOP interspinous implant: 1-year results Eur Spine J 2004 13 22 31 10.1007/s00586-003-0581-4 14685830 PMC3468027

[B10] Zucherman JF Hsu KY Hartjen CA Mehalic TF Implicito DA Martin MJ Johnson DR 2nd Skidmore GA Vessa PP Dwyer JW Puccio ST Cauthen JC Ozuna RM A multicenter, prospective, randomized trial evaluating the X STOP interspinous process decompression system for the treatment of neurogenic intermittent claudication: two-year follow-up results Spine 2005 30 1351 1358 10.1097/01.brs.0000166618.42749.d1 15959362

[B11] Szpalski M Gunzburg R Lumbar spinal stenosis in the elderly: an overview Eur Spine J 2003 12 Suppl 2 S170 175 13680315 10.1007/s00586-003-0612-1 PMC3591819

[B12] Hamanishi C Matukura N Fujita M Tomihara M Tanaka S Cross-sectional area of the stenotic lumbar dural tube measured from the transverse views of magnetic resonance imaging J Spinal Disord 1994 7 388 393 7819638

[B13] Weishaupt D Schmid MR Zanetti M Boos N Romanowski B Kissling RO Dvorak J Hodler J Positional MR imaging of the lumbar spine: does it demonstrate nerve root compromise not visible at conventional MR imaging? Radiology 2000 215 247 253 10751495 10.1148/radiology.215.1.r00ap06247

[B14] Wildermuth S Zanetti M Duewell S Schmid MR Romanowski B Benini A Boni T Hodler J Lumbar spine: quantitative and qualitative assessment of positional (upright flexion and extension) MR imaging and myelography Radiology 1998 207 391 398 9577486 10.1148/radiology.207.2.9577486

[B15] Stucki G Daltroy L Liang MH Lipson SJ Fossel AH Katz JN Measurement properties of a self-administered outcome measure in lumbar spinal stenosis Spine 1996 21 796 803 10.1097/00007632-199604010-00004 8779009

[B16] Pizones J Izquierdo E Sanchez-Mariscal F Zuniga L Alvarez P Gomez-Rice A sequential damage assessment of the different components of the Posterior Ligament Complex (PLC) after MRI interpretation: prospective study 74 traumatic fractures Spine 2012 37 E662 E667 10.1097/BRS.0b013e3182422b2b 22146288

[B17] Kondrashov DG Hannibal M Hsu KY Zucherman JF Interspinous process decompression with the X-STOP device for lumbar spinal stenosis: a 4-year follow-up study J Spinal Disord Tech 2006 19 323 327 10.1097/01.bsd.0000211294.67508.3b 16826002

[B18] Richards JC Majumdar S Lindsey DP Beaupre GS Yerby SA The treatment mechanism of an interspinous process implant for lumbar neurogenic intermittent claudication Spine 2005 30 744 749 10.1097/01.brs.0000157483.28505.e3 15803075

[B19] Wiseman CM Lindsey DP Fredrick AD Yerby SA The effect of an interspinous process implant on facet loading during extension Spine 2005 30 903 907 10.1097/01.brs.0000158876.51771.f8 15834334

[B20] Swanson KE Lindsey DP Hsu KY Zucherman JF Yerby SA The effects of an interspinous implant on intervertebral disc pressures Spine 2003 28 26 32 10.1097/00007632-200301010-00008 12544951

[B21] Fuchs PD Lindsey DP Hsu KY Zucherman JF Yerby SA The use of an interspinous implant in conjunction with a graded facetectomy procedure Spine 2005 30 1266 1272 discussion 1273–1264 10.1097/01.brs.0000164152.32734.d2 15928550

[B22] Kuchta J Sobottke R Eysel P Simons P Two-year results of interspinous spacer (X-Stop) implantation in 175 patients with neurologic intermittent claudication due to lumbar spinal stenosis Eur Spine J 2009 18 823 829 10.1007/s00586-009-0967-z 19387698 PMC2899666

[B23] Lee J Hida K Seki T Iwasaki Y Minoru A An interspinous process distractor (X STOP) for lumbar spinal stenosis in elderly patients: preliminary experiences in 10 consecutive cases J Spinal Disord Tech 2004 17 72 77 discussion 78 10.1097/00024720-200402000-00013 14734979

[B24] Siddiqui M Smith FW Wardlaw D One-year results of X Stop interspinous implant for the treatment of lumbar spinal stenosis Spine 2007 32 1345 1348 10.1097/BRS.0b013e31805b7694 17515824

[B25] Kim DH Tantorski M Shaw J Martha J Li L Shanti N Rencu T Parazin S Kwon B Occult spinous process fractures associated with interspinous process spacers Spine 1976 36 E1080 E1085 Phila Pa 1976 21343860 10.1097/BRS.0b013e318204066a

[B26] Sobottke R Schluter-Brust K Kaulhausen T Rollinghoff M Joswig B Stutzer H Eysel P Simons P Kuchta J Interspinous implants (X Stop, Wallis, Diam) for the treatment of LSS: is there a correlation between radiological parameters and clinical outcome? Eur Spine J 2009 18 1494 1503 10.1007/s00586-009-1081-y 19562386 PMC2899374

